# Use of Daily Patient-Reported Outcome Measurements in Pediatric Cancer Care

**DOI:** 10.1001/jamanetworkopen.2022.23701

**Published:** 2022-07-26

**Authors:** Andreas Meryk, Gabriele Kropshofer, Benjamin Hetzer, David Riedl, Jens Lehmann, Gerhard Rumpold, Alexandra Haid, Verena Schneeberger-Carta, Bernhard Holzner, Roman Crazzolara

**Affiliations:** 1 Department of Pediatrics, Medical University of Innsbruck, Innsbruck, Austria; 2 Department of Psychiatry, Psychotherapy and Psychosomatics, University Hospital of Medical Psychology, Medical University of Innsbruck, Innsbruck, Austria; 3 Department of Psychiatry, Psychotherapy and Psychosomatics, University Hospital of Psychiatry II, Medical University of Innsbruck, Innsbruck, Austria

## Abstract

**Importance:**

Patient-reported outcome measurements (PROMs) are emerging as an important component of adult cancer care, but little has been done with regard to PROMs for pediatric cancer care.

**Objectives:**

To identify pediatric patients with cancer who are at risk of severe adverse effects of treatment and provide individualized supportive care using PROMs.

**Design, Setting, and Participants:**

This single-center cohort study with PROMs implemented in daily clinical routine was conducted from May 1, 2020, to November 15, 2021, among pediatric patients with a cancer diagnosis or their proxies. Inclusion criteria were treatment with chemotherapy and at least 30 days of active participation. Patients were followed up until completion of therapy or through ongoing therapy until November 15, 2021; data were analyzed from November 15, 2021, through January 31, 2022.

**Exposures:**

Cancer treatment, including chemotherapy, surgery, and radiotherapy.

**Main Outcomes and Measures:**

The primary outcome was occurrence and severity of ubiquitous complications of cancer treatment, such as nausea, appetite loss, pain, sleep disturbance, and deterioration of physical functioning. The secondary outcome was the identification of early and appropriate clinical interventions based on detection of cancer-related symptoms via PROMs.

**Results:**

A total of 4410 daily PROMs from 7082 therapy days for 40 children (35 children aged 5-18 years and 5 proxies for children aged 1-4 years) (median age, 9.1 [IQR, 6.3-12.2] years; 26 [65.0%] male) were analyzed during a median follow-up of 145.5 (IQR, 103.8-244.5) days. All participants were White. The overall median completion rate was 60.1% (IQR, 37.9%-81.0%); this rate was slightly lower during home care vs inpatient stay (57.5% [IQR, 30.7%-85.9%] vs 65.0% [IQR, 49.6%-92.5%], respectively; *P* = .01), with a decreasing trend over time (65.6% [IQR, 51.6%-85.9%] for the first 90 days vs 42.9% [IQR, 29.3%-82.3%] for beyond 90 days; *P* < .001). Severe symptoms were reported on 657 days (14.9%); most symptoms were associated with physical functioning, followed by pain, sleep disturbance, and nausea and appetite loss. In total, 321 adverse events (AEs) and cases of health deterioration were documented, and PROMs were completed for 251 (78.2%) of these events. Across all AEs, self-reported pain was the most useful marker, particularly when analyzed on the day before onset, and was associated with an odds ratio of 3.65 (95% CI, 1.54-8.62; *P* = .005) for the presence of mucositis.

**Conclusions and Relevance:**

The findings of this cohort study suggest that PROMs reflect daily symptoms in pediatric patients with cancer and assist in clinical management and intervention for AEs.

## Key Points

**Question:**

Can daily patient-reported outcome measurements (PROMs) be integrated into routine and supportive care for children with cancer?

**Findings:**

This cohort study included 40 pediatric patients with cancer with 7082 therapy days who completed 4410 daily PROMs during a median follow-up of 145.5 days, yielding a completion rate of 60.1%. The most common moderate or severe symptom reported was physical functioning, followed by pain, sleep disturbance, and nausea and appetite loss.

**Meaning:**

The findings of this cohort study suggest that completion of daily PROMs by pediatric patients with cancer can assist in their clinical management, identify adverse events, and help determine requisite medical interventions.

## Teaser

This cohort study examines the use of daily web-based patient-reported outcome measurements by pediatric patients with cancer to assess their health status and identify adverse effects of treatment.

## Introduction

Digital health care is a broad concept that includes the use of diverse technologies for mobile health, health information, and wearable devices to quantify the mental and physical condition of the user. One example of this concept is the use of patient-reported outcome measurements (PROMs) for supportive care among adults with cancer.^1,2^ When PROMs are recorded, subjective impairments caused by the disease and its treatment are identified early and medical interventions are initiated, which results in improved overall survival and health-related quality of life.^1,2,3,4,5^ Although the use of PROMs in clinical cancer research has repeatedly been encouraged by regulatory agencies, implementation has primarily been restricted to adult patients. In fact, less than 1% of pediatric clinical trials submitted to ClinicalTrials.gov and EudraCT (European Union Drug Regulating Authorities Clinical Trials) use PROMs as a primary end point.^6,7^ Several shortfalls in pediatrics may be explained in part by (1) insecurity regarding the choice of the appropriate PROM questionnaire, (2) skepticism regarding PROM data collection, (3) inappropriate timing of assessment, and (4) inadequate statistical analysis and outcome interpretation.^8^ The biggest hurdle to overcome in pediatrics, however, is the fact that the professional team needs to interact not only with the children, but also with their proxies. Although some PROMs offer promise in routine clinical care of pediatric patients with cancer,^9,10,11^ no study to our knowledge has evaluated daily PROMs on a long-term basis or differentiated between surveys administered in the hospital or at home.

Our group^12^ recently developed a unique, web-based approach for daily child self-reporting and parent-based proxy reporting (ePROtect) and demonstrated its feasibility for implementation in clinical care. We have now prospectively enrolled a larger number of patients in an effort to better understand the utility of patients’ health status measurement. We hypothesized that patients and their proxies would thoroughly report symptoms concerning their illness, irrespective of their stay, and that their information would help the professional health care team to better control their symptoms.

## Methods

### Participants

This single-center cohort study was conducted from May 1, 2020, to November 15, 2021, at the Medical University of Innsbruck. Patients were followed up until completion of cancer therapy or during ongoing therapy until November 15, 2021. All German-speaking children and adolescents with cancer who were age 1 to 18 years at enrollment and their primary caregivers were recruited for the study. Start of chemotherapy within 15 days of diagnosis and at least 30 days of active ePROtect participation (completion of 30 daily PROMs) constituted inclusion criteria. Patients who consented to participate and did not use ePROtect were listed as dropouts (ie, loss of interest in continuing the study). Exclusion criteria were cognitive disability or visual impairment that precluded use of the web application. The ethics committee at the Medical University of Innsbruck approved this study; written informed consent was obtained from all children 5 years and older and from the caregivers of all children. The study followed the Strengthening the Reporting of Observational Studies in Epidemiology (STROBE) reporting guideline.

### Study Design

Children and adolescents aged 5 to 18 years and proxies for children aged 1 to 4 years were approached and trained in the use of the ePROtect software by the professional health care team (A.M., G.K., B.H., A.H., and R.C.). A description of the ePROtect software and its use in daily clinical care has been published previously.^12^ Patients were instructed to complete the symptom monitoring once per day during the entire treatment period. If a patient did not have a mobile device, the health care team provided an iPad (Apple Inc) during the patient’s hospital stay. Symptom monitoring was checked daily before the morning round; in the case of relevant deviations (symptom score ≤50 of 100 possible), the results were discussed immediately at the patient’s bedside. If the patient and the caregiver were not in the hospital, they were instructed to complete the questionnaire as if they were hospitalized. Oversight was provided daily by the medical team, and if the symptom score was 50 or less, the patients or their caregivers were immediately called by the physicians to confirm the reported symptoms, discuss the situation, and initiate an intervention if needed.

### Measurement Tools and Assessment

The PROMs described herein were initially introduced and evaluated in our previous study.^12^ These PROMs monitor patients’ symptom burden during and after treatment and were selected in an eminence-based approach involving a team of different clinicians and patient-reported outcome researchers (eMethods in the Supplement provides more information on the development process). The PROMs in ePROtect are implemented via 6 daily questions allocated to 4 domains: pain (1 item), nausea and appetite loss (2 items), physical functioning (2 items), and sleep disturbance (1 item) (eMethods in the Supplement). In contrast to our previous study,^12^ questions regarding cognitive impairment were omitted because scores were generally high and not responsive to change, and the perceived clinical value of those 2 items was deemed low by the team. Each question’s recall period was the past 24 hours. The responses to the items were given on a scale with 3 faces for those younger than 8 years or on a 5-point Likert scale ranging from never to almost always for those 8 years and older. Answers were then summed for multiple-item scales and converted to a scale ranging from 0 to 100 using linear transformation (eMethods in the Supplement). For all scales, a lower score (closer to 0) indicated higher symptom burden, whereas a higher score (closer to 100) indicated less symptom burden. To support day-to-day clinical use and interpretation, scores were grouped in 3 symptom degrees: none to mild (51-100), moderate (26-50), and severe (0-25). These were color coded for health care professionals in the ePROtect software as green for mild, orange for moderate, and red for severe. Considering that we used a short and newly developed item list, thresholds were established based on clinical experience and findings from our previous studies.^12,13^ Moreover, symptom deterioration to a cutoff score of 50 or less was considered a significant and important difference that prompted clinical action.

### Data Collection

We collected patients’ sociodemographic and clinical data at study inclusion. In addition, routine physical examination and laboratory checks with regular determination of complete blood cell count and measurements of C-reactive protein, interleukin 6, procalcitonin, aspartate transaminase, alanine transaminase, and bilirubin levels were performed at hospital admission (ambulatory care and inpatient stay). Based on Common Terminology Criteria for Adverse Events, version 5, the following adverse event (AE) terms were graded for this study: febrile neutropenia, mucositis, pneumonia, and severity of anemia and hepatopathy. If an AE was not included in this classification, it was documented as *other* in the case report form.

### Interventions

When febrile neutropenia, pneumonia, mucositis, pain, nausea, or sleep disturbances occurred, we applied standardized interventions according to institutional protocols and following the recommendations of guidelines on how to treat these conditions.^14,15,16,17^ In the case of deviations in the PROM score to 50 or less, the telephone contact or the bedside discussion with the patient or caregiver was documented. Subsequently, the type of intervention—whether medical advice or recommendation for drug therapy—or immediate presentation with admission to the ward was documented.

### Outcome Measurements

Our primary outcome was the quantification of frequency and severity of patient self-reported, cancer-specific complications, including pain, nausea and appetite loss, sleep disturbance, and deterioration of physical functioning. The secondary outcomes were the analysis of PROM completion rate, the association with typical cancer-related AEs, and the identification of early and appropriate clinical interventions after detection of cancer-related symptoms via PROMs.

### Statistical Analysis

The data extraction date was November 15, 2021. Data were analyzed from extraction to January 31, 2022. Sample characteristics were calculated as absolute numbers, percentages, medians, means, and IQRs. The completion rate was calculated by dividing the number of days on which PROMs were completed by the number of days that the patient stayed in the study. Separate analyses were performed for treatment settings. *Inpatient* referred to admission to the hospital for a planned overnight stay, whereas *unplanned* was used for the treatment of a complication. Admission to the *pediatric intensive care unit* was indicated for serious treatment-related AEs. *Ambulatory care* defined any service or treatment that did not require hospitalization. In all inpatient situations, the health care team kept the patients at the hospital to monitor them closely, including physical examination and blood examination. With this in mind, the term *outpatient* referred to a stay outside the hospital without primary contact with any health care member.

Paired and unpaired *t* tests were applied to analyze the distribution of frequency in the completion rates and deviations in symptom mean scores. A χ^2^ test was used to calculate the odds ratio significance. Differences were considered statistically significant at 2-sided *P* < .05 (Mann-Whitney *U* test), and Bonferroni correction was used to correct for multiple comparisons. All statistical analyses were performed using SPSS, version 26.0 (IBM Corporation). For data visualization, Prism, version 8.4 (GraphPad), was used.

## Results

### Patient Characteristics

Forty-eight children and adolescents who were first diagnosed with cancer between May 1, 2020, and October 15, 2021, were considered eligible for the study. Three children did not receive chemotherapy, and 5 patients dropped out within the first month (eFigure in the Supplement). Forty patients, including 5 represented by caregivers, actively participated in ePROtect, and the median follow-up period was 145.5 (IQR, 103.8-244.5) days. All participants included in this study were White. Patients had a median age of 9.1 (IQR, 6.3-12.2) years; 14 (35.0%) were female and 26 (65.0%) were male. Several different oncologic diagnoses were included (Table 1). All patients received standard induction chemotherapy, including 11 (27.5%) with surgical tumor removal and 4 (10.0%) with both surgery and subsequent radiotherapy. At the time of data analysis, 19 patients completed therapy; in 20, therapy was ongoing; and 1 died following a relapse at 147 days after initiation of therapy.

**Table 1.** Demographic and Clinical Characteristics of the Study Cohort

Characteristic	Patient data (N = 40)^a^
Age, median (IQR), y	9.1 (6.3-12.2)
Sex	
Female	14 (35.0)
Male	26 (65.0)
Underlying diagnosis	
ALL	13 (32.5)
AML	1 (2.5)
Hodgkin lymphoma	5 (12.5)
NHL	4 (10.0)
CNS tumor	6 (15.0)
STS	4 (10.0)
Others	7 (17.5)
Treatment	
Chemotherapy	25 (62.5)
Chemotherapy plus surgery	11 (27.5)
Chemotherapy plus surgery plus radiotherapy	4 (10.0)

Abbreviations: ALL, acute lymphoblastic leukemia; AML, acute myeloblastic leukemia; CNS, central nervous system; NHL, non–Hodgkin lymphoma; STS, soft tissue sarcoma.

^a^ Unless indicated otherwise, data are expressed as No. (%) of patients.

### Patient-Reported Outcome Measurements

**Figure 1.**
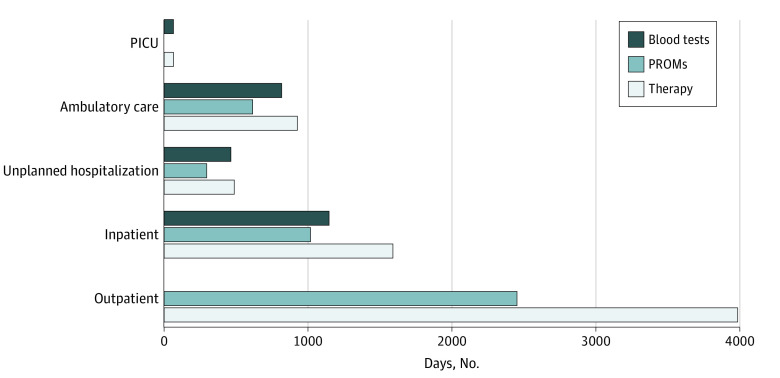
Allocation of Days During Cancer Treatment for Different Patient Settings Numbers of total therapy days, days with blood tests, and patient-reported outcome measurement (PROMs) assessments are depicted. Among treatment settings, inpatient refers to admission to the hospital for a planned overnight stay, whereas unplanned refers to treatment of a complication. Admission to the pediatric intensive care unit (PICU) is indicated by serious treatment-related adverse events. Ambulatory care defines any service or treatment that does not require hospitalization. Outpatient refers to a stay outside the hospital in home care.

The patients completed 4410 daily questionnaires on 7082 therapy days, corresponding to an overall median fulfillment of 60.1% (IQR, 37.9%-81.0%). The patients spent most of their time at home (outpatient, 3992 days [56.4%]) followed by planned hospitalization for chemotherapy (inpatient, 1595 days [22.5%]), ambulatory care (933 days [13.2%]), unplanned hospitalization (493 days [7.0%]), and the pediatric intensive care unit (69 days [1.0%]), the latter being caused by serious treatment-related AEs. Most PROM assessments were completed during outpatient stays (n = 2454) (eTable 1 in the Supplement), whereas a slightly higher median completion rate was achieved during inpatient compared with outpatient stays (65.0% [IQR, 49.6%-92.5%] vs 57.5% [IQR, 30.7%-85.9%]; *P* = .01) (eTable 1 in the Supplement). No significant differences in the median completion rate were seen among 3 different age groups (77.1% [IQR, 64.4%-89.3%] for patients aged 1-4 years; 48.6% [IQR, 37.9%-82.9%] for those aged 5-7 years; and 58.5% [IQR, 32.5%-74.1%] for those aged 8-18 years) (eTable 2 in the Supplement). However, patient adherence with self-reporting decreased significantly beyond day 90 of therapy from 65.6% (IQR, 51.6%-85.9%) to 42.9% (IQR, 29.3%-82.3%) (eTable 3 in the Supplement). Considering that blood tests are the most common parameter to assess the health condition, we compared their frequency with the constancy of daily PROMs. Blood tests were most commonly performed during pediatric intensive care unit stay (100% [IQR, 93.3%-100%]), followed by unplanned hospitalization (100% [IQR, 97.9%-100%]), ambulatory care (92.8% [IQR, 84.2%-100%]), and inpatient stay (67.0% [IQR, 51.8%-79.8%]) (Figure 1 and eTable 1 in the Supplement). In contrast, PROMs provided detailed health information during outpatient stays (57.5% [IQR, 30.7%-85.9%] of possible days) (eTable 1 in the Supplement), during which blood tests were not performed.

### PROM Scoring

A total of 17 620 individual symptoms were self-reported to the health care team and assigned to 4 different categories: pain, nausea and appetite loss, physical functioning, and sleep disturbance. The mean scores were 83.0 (IQR, 70.5-94.9) for pain, 83.0 (IQR, 78.6-91.8) for nausea and appetite loss, 75.7 (IQR, 65.2-90.5) for physical functioning, and 89.3 (IQR, 84.6-97.8) for sleep disturbance (eTable 4 in the Supplement). Among these, 3080 entries (17.5%) on 657 days (14.9%) were of moderate (score, 26-50) or severe (score, 0-25) intensity. The most common moderate or severe symptom was decreased physical functioning (1074 of 4404 [24.4%]), followed by pain (1035 of 4412 [23.5%]), sleep disturbance (493 of 4407 [11.2%]), and augmented nausea and appetite loss (478 of 4397 [10.9%]). Patients were routinely examined with a standardized approach, resulting in the detection of 321 AEs and cases of health deterioration. Because ePROtect triggered an automated alarm, when a symptom severity of 50 or less was reported for the first time, PROM alerts detected the occurrence of AEs and health deterioration in 251 cases (78.2%), whereas for 70 events (21.8%), PROMs were missing. Of the 251 PROMs associated with health deterioration and AEs, symptom monitoring anticipated interventions by the health care team in 147 (58.6%). In detail, this led to immediate counsel for symptom management (74 [50.3%]), supportive initiation or change of medication regimen (51 [34.7%]), or referral to the emergency department or hospital (22 [15.0%]). Among the remaining 104 of the 251 events (41.4%), intervention was not possible owing to missing changes in self-reported health status in 90 (86.5%). Overall, for all individual symptom categories, patients reported significantly higher symptom burden in cases of unplanned compared with planned admission (eg, mean difference for pain, −14.5 [95% CI, −22.9 to −6.0] vs 2.2 [95% CI, −1.0 to 5.3]; *P* < .001), particularly 1 day before admission (Table 2).

**Table 2.** Mean Scores and Mean Differences for Planned and Unplanned Hospitalizations 1 Day Before and on the Day of Admission^a^

Domain	Planned admission (n = 105)			Unplanned admission (n = 38)		
	Mean score (IQR)		Mean difference (95% CI)	Mean score (IQR)		Mean difference (95% CI)
	Day −1	Day 0		Day −1	Day 0	
Individual						
Pain	88.1 (75.0 to 100)	90.0 (87.5 to 100)	2.2 (−1.0 to 5.3)^b^	67.8 (50.0 to 100)	53.3 (25.0 to 100)	−14.5 (−22.9 to −6.0)^b^
Nausea and appetite loss	88.0 (75.0 to 100)	87.1 (75.0 to 100)	−0.8 (−2.3 to 0.6)^c^	74.0 (62.5 to 100)	67.4 (50.0 to 87.5)	−6.6 (−12.6 to −0.6)^c^
Physical functioning	83.6 (75.0 to 100)	84.3 (75.0 to 100)	0.8 (−0.7 to 2.4)^d^	59.2 (34.4 to 78.1)	50.0 (25.0 to 75.0)	−9.2 (−16.5 to −1.9)^d^
Sleep disturbance	92.7 (100 to 100)	92.6 (100 to 100)	−0.3 (−3.5 to 2.9)^e^	76.3 (50.0 to 100)	63.8 (25.0 to 100)	−12.5 (−25.1 to 0.1)e^d^
Global	87.9 (81.3 to 100)	88.4 (81.3 to 100)	0.4 (−0.8 to 1.7)^b^	69.3 (50.0 to 87.5)	58.6 (43.8 to 82.0)	−10.7 (−16.0 to −5.4)^b^

^a^ Data of 30 patients with planned admissions and 20 patients with unplanned admissions were analyzed. *P* values were calculated using Mann-Whitney *U* test for comparison of mean differences between planned and unplanned admissions.

^b^ *P* < .001.

^c^ *P* = .03.

^d^ *P* < .001.

^e^ *P* = .001.

### Association of PROM Score With Adverse Events

**Figure 2.**
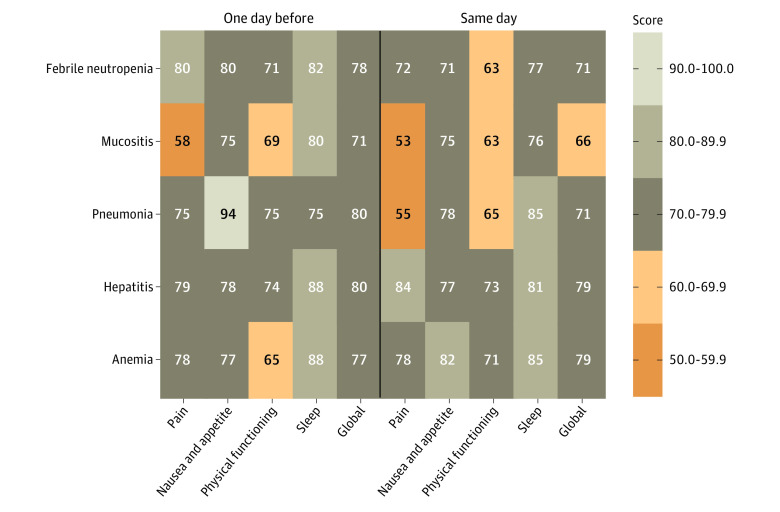
Association of Symptom Scores With 5 Adverse Events For the following 128 adverse events, a patient-reported outcome measurement (PROM) entry was made: anemia (n = 43), febrile neutropenia (n = 34), mucositis (n = 28), hepatitis (n = 18), and pneumonia (n = 5). Mean symptom scores for the domains of pain, nausea and appetite loss, physical functioning, and sleep disturbance and a total score are shown. Lower scores indicate greater symptom severity.

Referring to the baseline values of health-related quality of life across all classifications (eTable 4 in the Supplement), we analyzed PROM scores 1 day before and on occurrence of the 5 most common AEs: anemia (n = 43), febrile neutropenia (n = 34), mucositis (n = 28), hepatitis (n = 18), and pneumonia (n = 5). Focusing on the highest symptom burden, strong pain was reported in cases of mucositis (mean [SEM] score, 53.0 [6.5] points) and pneumonia (mean [SEM] score, 55.0 [12.2] points) (day of occurrence) (Figure 2). More importantly, 1 day before the clinical manifestation, pain was reported with the highest symptom burden for mucositis (mean [SEM] score, 58.0 [5.6] points) (Figure 2), and a low pain score (≤50) was significantly associated with this AE (odds ratio, 3.65 [95% CI, 1.54-8.62]; *P* = .005) (Table 3).

**Table 3.** Association of Pain Score of 50 or Less With Adverse Events 1 Day Before Clinical Manifestation^a^

Adverse event	Odds ratio (95% CI)
Febrile neutropenia	0.58 (0.26-1.33)
Mucositis	3.65 (1.54-8.62)^b^
Pneumonia	1.59 (0.22-11.61)
Hepatitis	0.76 (0.29-2.15)
Anemia	0.68 (0.32-1.43)

^a^ Scores range from 0 to 100, with lower scores indicating more severe pain.

^b^ Significant at *P* = .005 with Bonferroni-adjusted α value.

## Discussion

In recent years, many new models and technologies have improved patient participation in health care.^1,2,3,4,18,19,20,21^ For metastatic breast, lung, genitourinary, and gynecologic tumors, adult patients allocated to standardized symptom assessment develop complications less frequently and improve treatment compliance and quality of life; more importantly, these patients have better overall survival compared with patients allocated to usual care.^1,2,3,4,5^ The fact that patient self-reporting offers such a great potential is diametrically opposed to its very sparse use, which can be generalized for patients with cancer,^18^ but applies more in the pediatric population with cancer,^6^ among whom high symptom burden develops and, if not consistently met with intervention, leads to indeterminate long-term consequences.^22^

According to the EUROCARE Working Group,^23^ approximately 16 000 cases of cancer are diagnosed in children younger than 15 years every year, compared with 2.7 million in adults. In this context, the resources in childhood cancer have supported collaborative group clinical research, which has successfully enrolled enough patients in large multicenter trials and translated the tools of molecular genetics to improve the cure rate to more than 80%.^24^ However, the research interest has not sufficiently focused on secondary aims of clinical trials, such as the assessment of physical factors (eg, pain, nausea, fatigue) or emotional and social factors (eg, fear of relapse, separation from the family), circumstances that can be summarized under the term *health-related quality of life*.^25,26^ General barriers for implementation may include insecurity regarding the choice of the appropriate tools for assessment of symptom reporting, skepticism regarding the usefulness of data collection, communication barriers (eg, if not transferred electronically), inadequate statistical analysis, and outcome misinterpretation.^8^ Finally, the health care relationship in pediatrics is a triad in which the caregivers are intimately involved in patient management and definitely need to be involved in symptom control, and access to the child must be age appropriate.^27,28^

When introducing ePROtect, we carefully identified the use of valid and reliable disease-specific PROMs with high relevance for children’s health-related quality of life: appetite loss, fatigue, nausea, pain, physical functioning, and sleep disturbance.^12^ Notably, current practice in childhood cancer treatment includes the sequential administration of intravenous chemotherapy during a period of a few days spent in the hospital and most of the time including a recovery period, which is best spent at home in family care. Therefore, our system has to meet the demand for anytime and anywhere, particularly when the patient is sent home to recover.^12,13^ In our study, we found that patients can complete questionnaires on most days, irrespective of age, diagnosis, and therapy. Patient completion rates differ between the inpatient and outpatient modalities: the completion rate is highest during inpatient stay and slightly lower for outpatient stay, indicating good adherence to clinical contact. Nevertheless, patients complete the monitoring on more than 50% of the days, even during unplanned inpatient stays. To evaluate what a sufficient or minimal frequency of daily PROMs could be, we compared these with the number of blood tests performed. PROMs reached a similar frequency in the inpatient and outpatient settings as inpatient blood tests and provided regular and independent information on the clinical course of the patient. However, the slightly lower completion rate during outpatient stays together with a decreasing trend to ongoing therapy duration will necessitate incorporating tools to improve long-term participation in ePROtect (eg, gamification, push notification). Further in-depth analyses on the completion rates are warranted and currently in preparation. The importance of regular and continuous participation is evident, because the daily PROM assessment was missing in 70 cases of AEs and health deterioration (21.8%) and hampered medical intervention.

Because most days are spent by the patient at home (56.4%), it is important to investigate whether ePROtect enables early detection of health deterioration before clinical manifestation. Indeed, mean scores for the pain, nausea, physical activity, and sleep disturbance domains are 15 to 20 points lower in cases of unplanned compared with planned hospitalization 1 day before admission. When comparing the individual symptom scores for the 5 most common AEs, pain is seen to be the most important self-reported symptom with a significant predictive value for the onset of mucositis.

### Strengths and Limitations

The main strength of this study is the high adherence and participation rate in patients with diverse cancer. Limitations include the monocentric study design with a small sample size and that randomization or cross-sectional study design with and without access to ePROtect was currently not possible but will be considered when more centers are included. Nevertheless, our findings contribute to filling the knowledge gap in which pediatric self-reporting is not only possible but can also be integrated into clinical routine.

## Conclusions

The findings of this cohort study suggest that electronic symptom self-measuring with ePROtect is very well received for inpatient and outpatient use, can provide early detection of toxic effects, and can anticipate necessary admissions or medical interventions. In the context of increasing demands regarding patient self-advocacy and self-management, symptom self-reporting allows patients to participate more actively in their therapy and may help improve their quality of life. For health care professionals, ePROtect facilitates communication and awareness of symptoms, which can result in more efficient treatment of patients.

## Notes

Supplement. **eFigure.** Flowchart of Patient Enrollment **eTable 1.** Allocation of Therapy Days and the Corresponding Amount and Frequency of ePROtect Use and Hospital Blood Tests **eTable 2.** Participation in ePROtect for the 3 Age Groups **eTable 3.** Completion Rate of Assessments Within First 90 Days of Therapy and Beyond **eTable 4.** Mean Scores and Classification of Symptoms **eMethods.** ePROtect Questionnaire Development and Scoring Information
