# Supplementary material for: Use of Daily Patient-Reported Outcome Measurements in Pediatric Cancer Care
Source: JAMA Netw Open. 2022 Jul 26;5(7):e2223701. doi: 10.1001/jamanetworkopen.2022.23701 (PMC9327576; doi:10.1001/jamanetworkopen.2022.23701)
Supplement: Supplement. — eFigure. Flowchart of Patient Enrollment eTable 1. Allocation of Therapy Days and the Corresponding Amount and Frequency of ePROtect Use and Hospital Blood Tests eTable 2. Participation in ePROtect for the 3 Age Groups eTable 3. Completion Rate of Assessments Within First 90 Days of Therapy and Beyond eTable 4. Mean Scores and Classification of Symptoms eMethods. ePROtect Questionnaire Development and Scoring Information [file jamanetwopen-e2223701-s001.pdf]

Supplementary Online Content

Meryk A, Kropshofer G, Hetzer B, et al. Use of daily patient-reported outcome measurements in pediatric cancer care. *JAMA Netw Open*. 2022;5(7):e2223701. doi:10.1001/jamanetworkopen.2022.23701

eFigure. Flowchart of Patient Enrollment
eTable 1. Allocation of Therapy Days and the Corresponding Amount and Frequency of ePROtect Use and Hospital Blood Tests
eTable 2. Participation in ePROtect for the 3 Age Groups
eTable 3. Completion Rate of Assessments Within First 90 Days of Therapy and Beyond
eTable 4. Mean Scores and Classification of Symptoms
eMethods. ePROtect Questionnaire Development and Scoring Information

This supplementary material has been provided by the authors to give readers additional information about their work.

© 2022 Meryk A et al. *JAMA Network Open*.

eFigure. **Flowchart of Patient Enrollment**

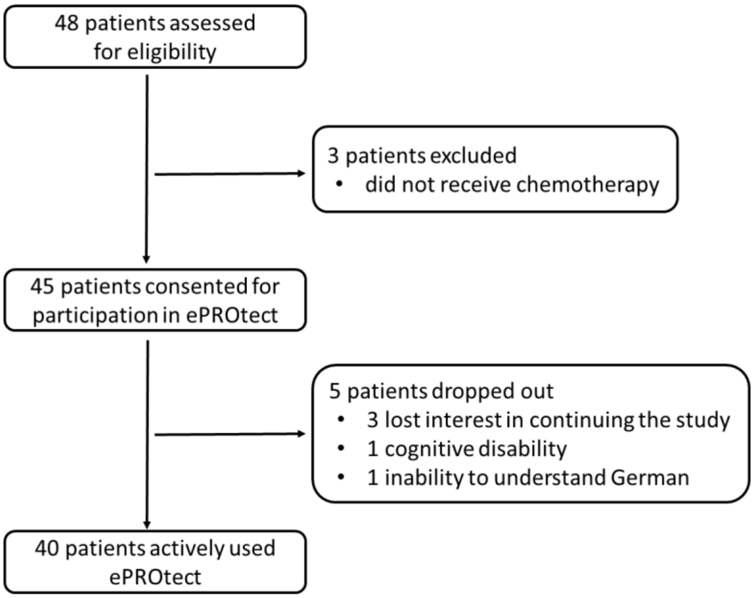

From May 2020 to November 2021, all oncologic paediatric and adolescent patients, who were below age 18 years were consecutively recruited. Three patients did not meet inclusion criteria as they did not receive a chemotherapy. 45 patients were introduced to the ePROtect software where they could complete PROMs daily online. Five patients dropped out within the first month. Of them, three lost interest in continuing the study, one showed cognitive disability due to underlying cancer and one had poor German language ability.

© 2022 Meryk A et al. *JAMA Network Open*.

eTable 1. Allocation of Therapy Days and the Corresponding Amount and Frequency of ePROtect Use and Hospital Blood Tests

	Outpatient	Inpatient	Unplanned hospitalization	Ambulatory care	PICU	Total
Therapy days (n)	3992	1595	493	933	69	7082
PROMs (n)	2454	1022	299	620	15	4410
Mean* (%)	58.4	68.0	61.6	54.8	28.5	60.9
Median* (%)	57.5	65.0	61.1	59.4	0	60.1
IQ1-IQ3* (%)	30.7-85.9	49.6-92.5	38.1-95.0	26.3-77.5	0-47.5	37.9-81.0
Min-Max (%)	11.1-100	21.4-100	12.5-100	5.0-100	0-100	26.2-98.7
Blood tests (n)	0	1152	470	816	67	2489
Mean* (%)	-	68.0	95.9	89.7	99.1	36.9
Median* (%)	-	67.0	100	92.8	100	33.2
IQ1-IQ3* (%)	-	51.8-79.8	97.9-100	84.2-100	100-100	19.0-51.8
Min-Max (%)	-	32.4-100	33.3-100	59.4-100	93.3-100	7.3-86.4

* analyzed for each patient separately and then calculated the mean, median and interquartile ranges of all patients

© 2022 Meryk A et al. *JAMA Network Open*.

eTable 2. Participation in ePROtect for the 3 Age Groups

	Total	outpatient	inpatient
Therapy days, mean			
1-4 years* (n=5)	162.2	85.4	35.4
5-7 years (n=13)	218.8	134.7	37.2
8-18 years (n=22)	155.8	82.5	42.5
ePROtect assessments, median %, (IQR)			
1-4 years*	77.1 (64.4-89.3)	90.9 (70.1-94.7)	89.7 (36.9-97.1)
5-7 years	48.6 (37.0-82.9)	43.0 (33.5-81.8)	64.0 (52.1-92.6)
8-18 years	58.5 (32.5-74.1)	55.0 (27.4-85.9)	66.9 (44.4-92.6)

* PROM assessment by proxy

© 2022 Meryk A et al. *JAMA Network Open*.

eTable 3. Completion Rate of Assessments Within First 90 Days of Therapy and Beyond

	Total	outpatient	inpatient
First 90 days of therapy (n=40)			
No. therapy days	2636	1635	1001
No. PROMs (ePROtect)	1781	1053	728
Mean participation ePROtect* (%)	66.4[a]	65.7[b]	73.8[c]
Median participation ePROtect* (%)	65.6	71.5	78.6
IQ1-IQ3 (participation ePROtect)* (%)	51.6-85.9	47.5-90.1	60.0-96.5
Beyond day 90th of therapy (n=34)			
No. therapy days	2951	2357	594
No. PROMs (ePROtect)	1695	1401	294
Mean participation ePROtect* (%)	52.3[a]	49.0[b]	56.0[c]
Median participation ePROtect* (%)	42.9	40.0	53.9
IQ1-IQ3 (participation ePROtect)* (%)	29.3-82.3	26.7-78.1	33.3-75.0

*analyzed for each patient separately and then calculated the mean, median and interquartile ranges of all patients. *P* value calculated using paired Student's t-test for comparison of participation between first 90 days and beyond day 90th: a $P < 0.001$, b $P = 0.002$,c $P < 0.001$

© 2022 Meryk A et al. *JAMA Network Open*.

eTable 4. Mean Scores and Classification of Symptoms

		Classification of Symptoms, n (%)		
**Domain**	**Mean Score (IQ1-IQ3)**	**None/Mild (Score 100-51)**	**Moderate (Score 50-26)**	**Severe (Score 25-0)**
Individual				
Pain	83.0 (70.5-94.9)	3377 (76.5%)	870 (19.7%)	165 (3.7%)
Nausea and appetite	83.0 (78.6-91.8)	3919 (89.1%)	368 (8.4%)	110 (2.5%)
Physical functioning	75.7 (65.2-90.5)	3330 (75.6%)	794 (18.0%)	280 (6.4%)
Sleep	89.3 (84.6-97.8)	3914 (88.8%)	369 (8.4%)	124 (2.8%)
Global	82.8 (74.5-90.5)	4159 (94.3%)	231 (5.2%)	22 (0.5%)

© 2022 Meryk A et al. *JAMA Network Open*.

eMethods. ePROtect Questionnaire Development and Scoring Information

Information on item selection and development process of the questionnaire used in ePROtect

The items for the ePROtect program were developed to monitor patient symptom burden during and after treatment. Initial development is described in our previous publication:

Meryk A, Kropshofer G, Hetzer B, et al. Implementation of daily patient-reported outcome measurements to support children with cancer. Pediatr Blood Cancer. 2021;68(11):e29279. doi:10.1002/pbc.29279

The goal was to develop a questionnaire covering key symptoms that appear during cancer therapy. The questionnaire was devised by an expert group consisting of clinicians and patient-reported outcome researchers following an eminence-based approach. Having a daily assessment meant that the questionnaire had to be short, hence no validated questionnaire such as the PedsQL could be used for daily monitoring. The expert group agreed to monitor the following key symptoms: physical functioning, nausea and appetite, pain, sleep and cognitive impairments (Table 1). Available response and correlated scores are shown in Figure 1 (<8 years) and Table 2 (>8 years). Items were constructed following research of items/issues assessed in similar questionnaires and discussion within the expert group until a final wording was agreed upon. Following the abovementioned implementation, the two questions regarding cognitive impairment were removed from the questionnaires as scores were generally high (i.e., scales did not seem to be sensitive to deterioration or change) and perceived clinical value of the two items was deemed low by the team. We therefore opted to remove the questions to keep the overall item burden low.

Daily questionnaire to monitor symptom burden

**German**	**English translation**	**Domain**
Hattest du gestern Probleme beim Schlafen?	Did you have trouble sleeping yesterday?	Sleep
Hattest du gestern Appetit?	Did you have an appetite yesterday?	Nausea and appetite
War dir gestern zu übel, um essen zu können?	Were you too sick to eat yesterday?	Nausea and appetite
Hattest du gestern Schmerzen?	Did you have pain yesterday?	Pain
War es gestern schwierig für dich zu gehen?	Was it difficult for you to walk yesterday?	Physical functioning
Fühltest du dich gestern zu müde zum Spielen?	Did you feel too tired to play yesterday?	Physical functioning
War es gestern schwierig für dich, in der Schule/im Kindergarten aufzupassen?	Was it difficult for you to pay attention at school/kindergarten yesterday?	Cognitive impairments
War es gestern schwierig für dich, dich daran zu erinnern, was dir jemand vorgelesen hat?	Was it difficult for you yesterday to remember what someone read to you?	Cognitive impairments

Both questions regarding cognitive impairments (grey colored) were removed from current version of our daily symptom monitoring.

© 2022 Meryk A et al. *JAMA Network Open*.

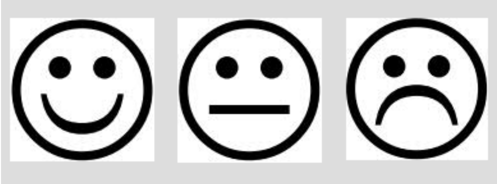

German	Nie	Manchmal	Fast immer
English	Never	sometimes	almost always
Points	0	2	4

Available responses for 5-7 year old patients

Available responses for 8-18 year old patients

**German**	**English translation**	**Points**
Nie	never	0
Fast nie	almost never	1
Manchmal	sometimes	2
Häufig	often	3
Fast immer	almost always	4

© 2022 Meryk A et al. *JAMA Network Open*.

Scoring information

For clinical use, scales are linearly converted to a 100–0 score wherein higher scores indicate lower symptom burden and lower scores indicate higher symptom burden.

For the questionnaire with smiley faces as answers, the happy face was coded with 0 points, the neutral face with 2 point, and the sad face with 4 points. For multi-items scales, a sum score was calculated. Scales were then converted to a 100–0 scale as follows:

$$Score = 100 - \frac{\frac{sum\ score}{number\ of\ items\ in\ the\ scale}}{4} \times 100$$

For the questionnaires answered on the 5-point Likert scale, where 0 = never, 1 = almost never, 2 = sometimes, 3 = often, and 4 = almost always. Score conversion was as follows:

$$Score = 100 - \frac{\frac{sum\ score}{number\ of\ items\ in\ the\ scale}}{4} \times 100$$

Important note: In our previous implementation study, we used a scaling form 0 to 4. However, we realized that a 100–0 score improves legibility and clarity (calculations with a scale from 0 to 4 would lead to decimals) and changed scaling.

© 2022 Meryk A et al. *JAMA Network Open*.
